# Supplementary figures and images for: cAMP−EPAC−PKCε−RIM1α signaling regulates presynaptic long-term potentiation and motor learning
Source: eLife. 2023 Apr 26;12:e80875. doi: 10.7554/eLife.80875 (PMC10171863; doi:10.7554/eLife.80875)

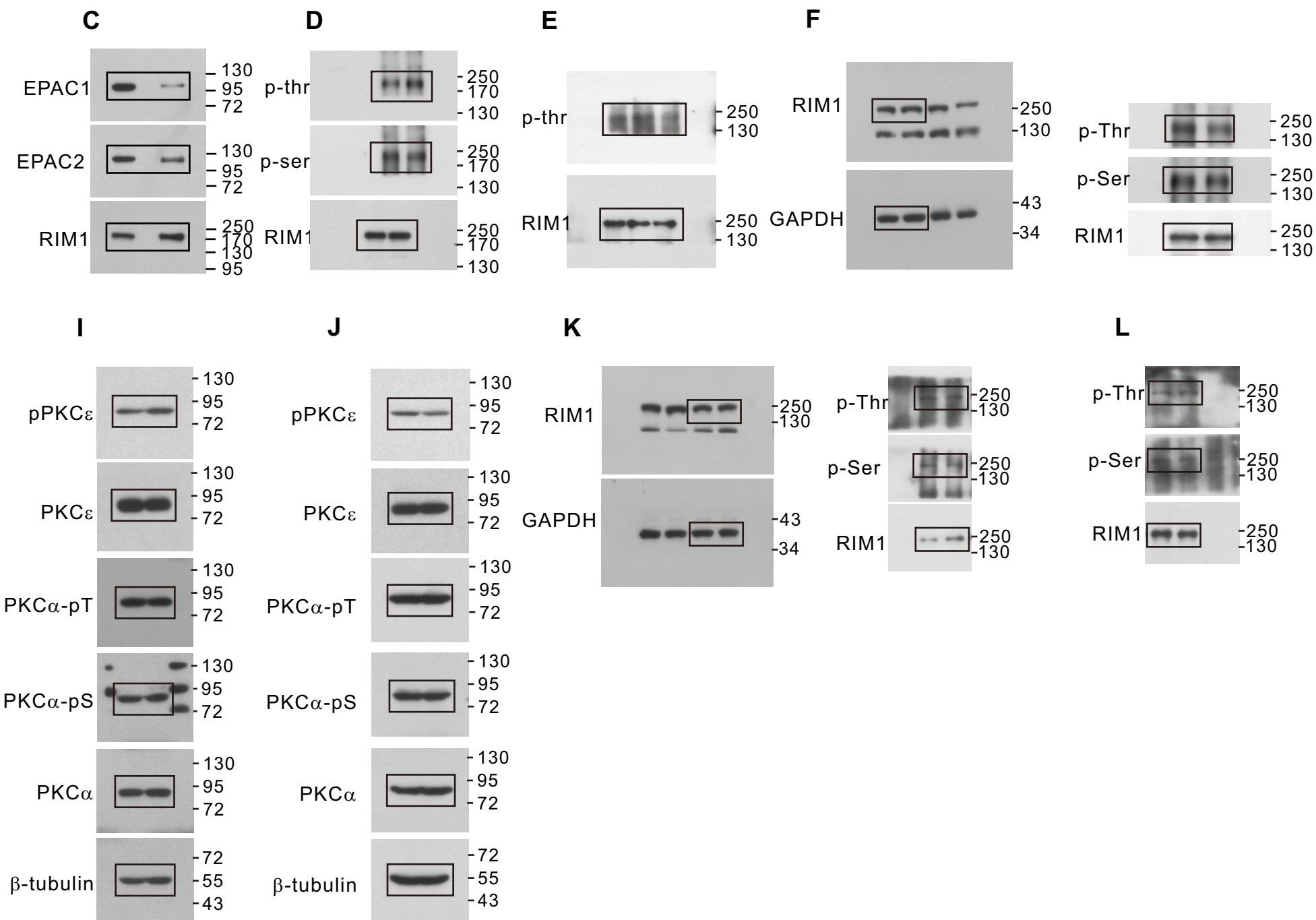

Supplement: Figure 1—source data 1. [file elife-80875-fig1-data1.pdf]

**B**

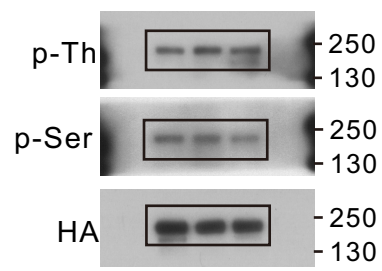

Supplement: Figure 1—figure supplement 2—source data 1. [file elife-80875-fig1-figsupp2-data1.pdf]

**F**

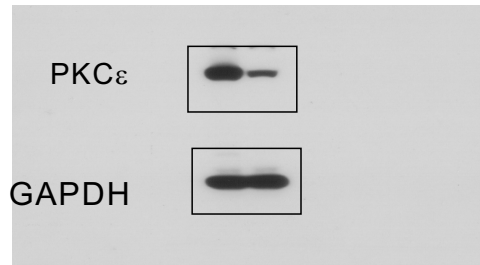

**E**

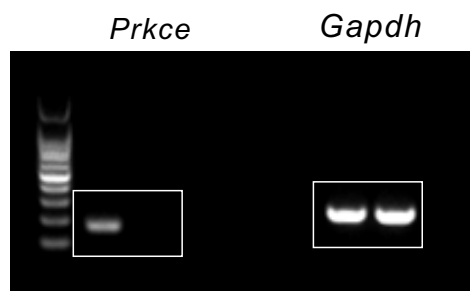

**J**

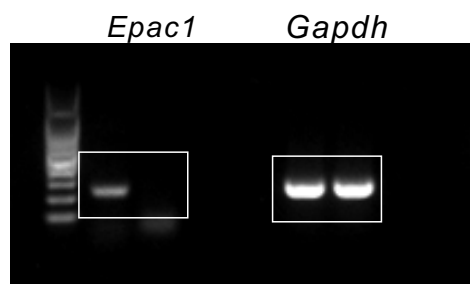

**L**

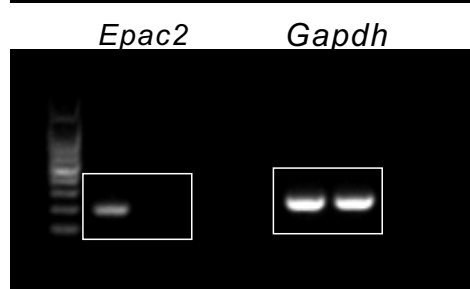

Supplement: Figure 1—figure supplement 3—source data 1. [file elife-80875-fig1-figsupp3-data1.pdf]

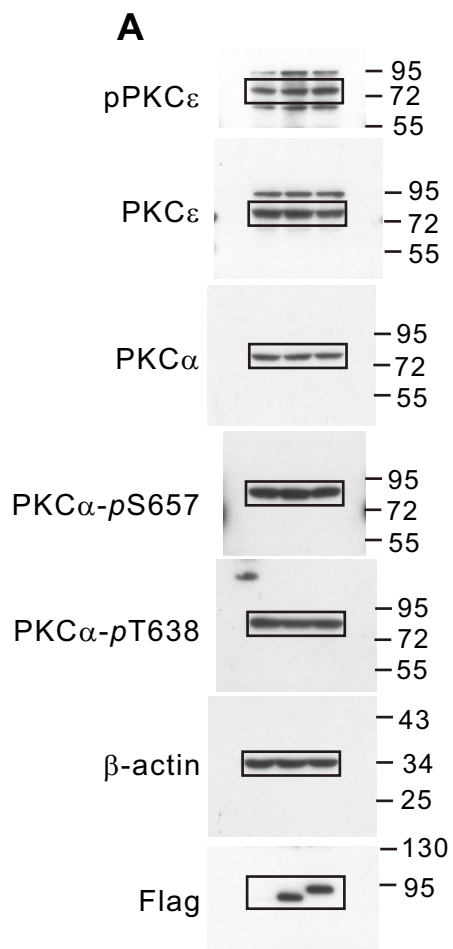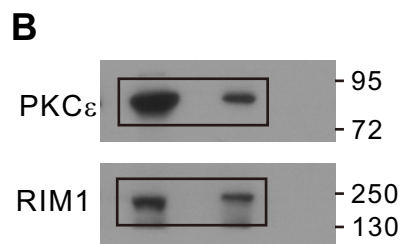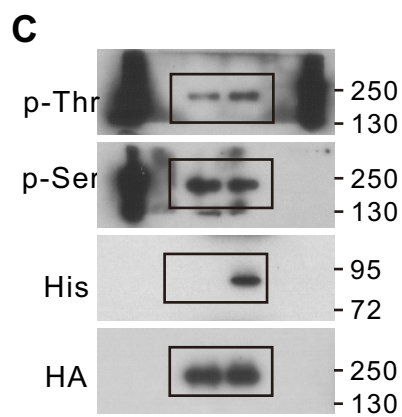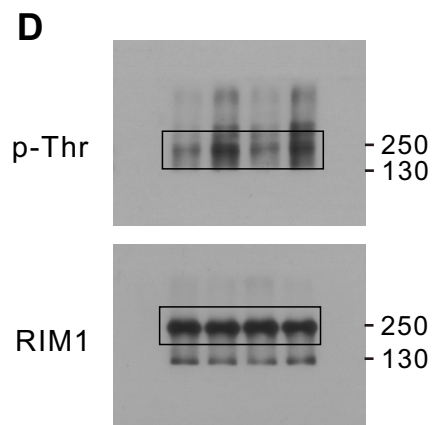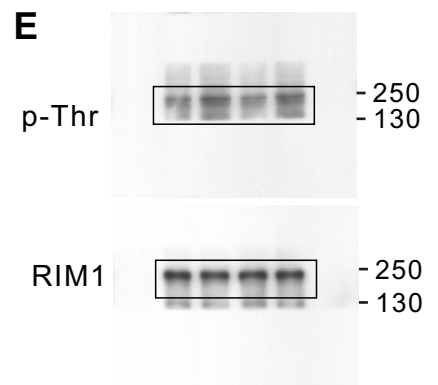

Supplement: Figure 1—figure supplement 4—source data 1. [file elife-80875-fig1-figsupp4-data1.pdf]

**G**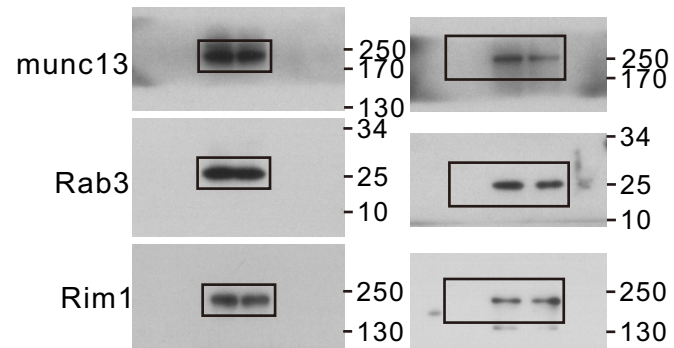**H**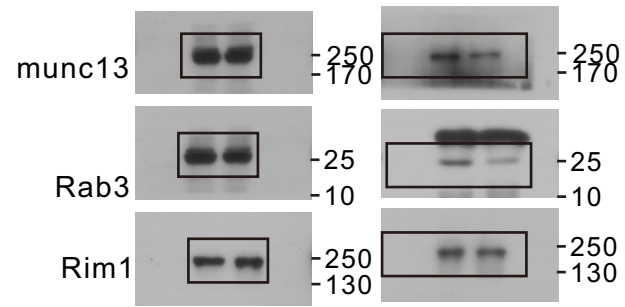**I**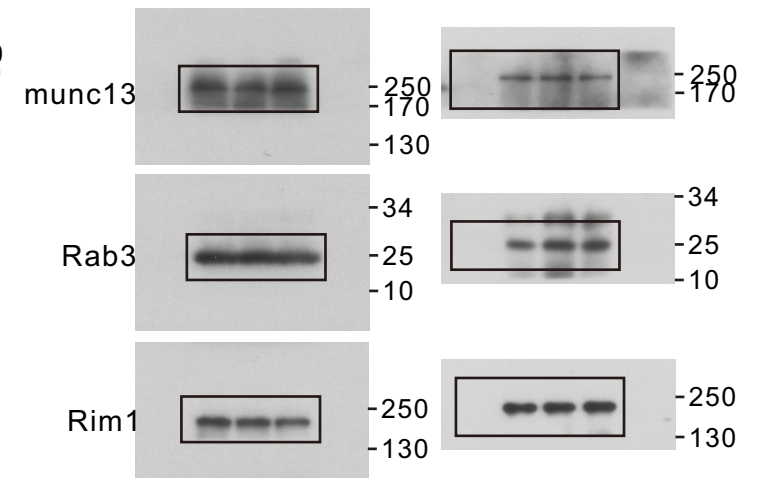**J**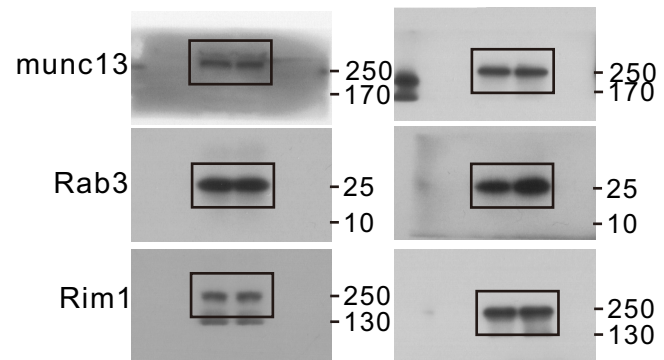**K**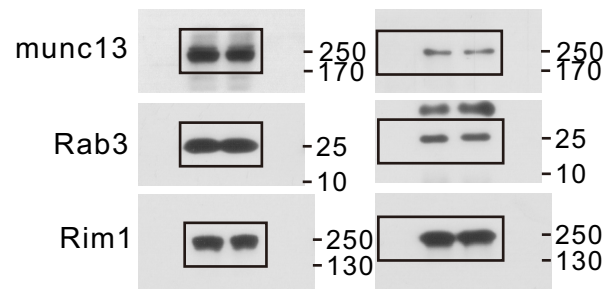

Supplement: Figure 2—source data 1. [file elife-80875-fig2-data1.pdf]
